# Supplementary material for: Identification of memory reactivation during sleep by EEG classification
Source: Neuroimage. 2018 Aug 1;176:203–14. doi: 10.1016/j.neuroimage.2018.04.029 (PMC5988689; doi:10.1016/j.neuroimage.2018.04.029)
Supplement: Manuscript Clean [file mmc1.docx]

**INERT INLINE SUPPLEMENTARY TABLE 1 HERE**


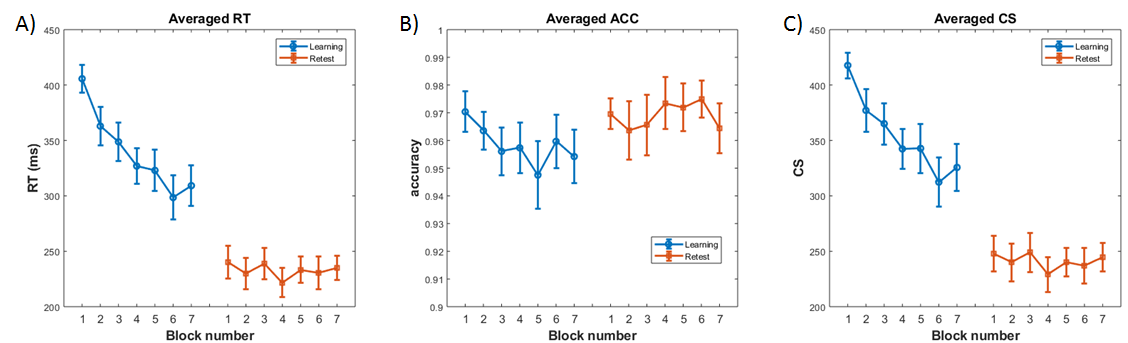


**Inline Supplemental Figure 1:** Learning curves for A) RT, B) Accuracy, and C) Composite score are plotted against block number. Blue represents pre-sleep training, while orange represents post-sleep testing.

**Inline Supplemental Figure 2**.

**
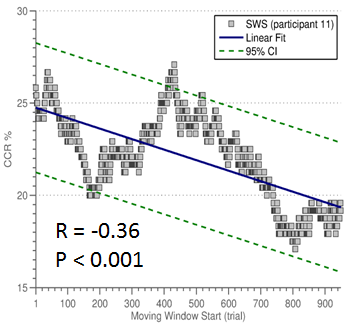
**

**Inline Supplemental Figure 2:** Representative plot of how classifier performance decreased across TMR repetitions. The CCR was calculated as the mean across 240 trials in a window that was slid forward in iterative steps. The x axis shows the trial number of this sliding window. The Y axis shows the mean CCR for the window.

**Inline Supplementary Table 1:** **Behavioural improvement.**

| **Initial learning** | **mean** | **sd** | **paired t-test** |
| --- | --- | --- | --- |
| **RT (ms)** | 96.44 | 60.60 | p < 0.001 |
| **Accuracy** | 0.02 | 0.03 | p = 0.093 |
| **CS** | 92.09 | 62.26 | p = 0.001 |
|  |  |  |  |
| **Overnight Improvement** | **mean** | **sd** | **paired t-test** |
| **RT (ms)** | -96.44 | 60.60 | p = 0.001 |
| **Accuracy** | -0.02 | 0.03 | p = 0.075 |
| **CS** | -92.09 | 62.26 | p = 0.001 |

**Inline Supplementary Table 1:** Behavioural improvements. The table shows values for improvement across initial pre-sleep learning and overnight improvement. Scores for reaction times (RT), Accuracy (Acc) and Composite Score (CS) are shown, along with standard deviations and p-values.
